# Fractionation of Saffron (*Crocus sativus* L.) Extract by Solid-Phase Extraction and Subsequent Encapsulation in Liposomes Prepared by Reverse-Phase Evaporation

**DOI:** 10.3390/molecules30224408

**Published:** 2025-11-14

**Authors:** Fabrizio Ruggieri, Maria Anna Maggi, Francesca Commito, Federica Badia, Luisa Giansanti

**Affiliations:** Department of Physical and Chemical Sciences (DSFC), University of L’Aquila, Via Vetoio (“A.C. De Meis” Building), 67100 L’Aquila, Italy; m.maggi@hortusnovus.it (M.A.M.); francesca.commito@graduate.univaq.it (F.C.);

**Keywords:** saffron extract, *Crocus sativus* L., crocins, liposomes, reverse-phase evaporation, encapsulation efficiency, solid-phase extraction, HPLC quantification

## Abstract

Saffron (*Crocus sativus* L.) is one of the most valued spices worldwide, rich in bioactive apocarotenoids such as crocins, picrocrocin, and safranal, which display antioxidant, neuroprotective, and anticancer properties. Saffron’s chemical composition is critical for its therapeutic efficacy and a combination of components appears essential to reach the best protection and increase tissue resilience, so stigmas were subjected to hydroalcoholic extraction followed by purification via solid-phase extraction to enriched crocin and picrocrocin fractions. The extracts were included in liposomes to enhance their bioavailability and gastrointestinal absorption by oral administration while protecting them in the harsh gastric environment, increasing their permeation and sustaining their release in the gastrointestinal tract. Liposomes were prepared by the reverse-phase evaporation method using saturated or unsaturated lipids extracted from soy. Encapsulation efficiency was determined by HPLC monitoring of *trans*-4GG crocin, *cis*-4GG crocin, and picrocrocin. The results indicate that liposomes show greater encapsulation capacity for hydrophilic apocarotenoids such as crocins (≈90% for *cis*-4GG, ≈50% for *trans*-4GG crocin) with respect to picrocrocins (<20%). These findings support the application of liposomal carriers to improve the stability, shelf-life, and potential bioavailability of saffron’s bioactive properties for nutraceutical, pharmaceutical, and functional food applications.

## 1. Introduction

Saffron (*Crocus sativus* L.) is universally recognized as one of the most valuable and costly spices, a status attributable not only to its labor-intensive cultivation and harvesting but also to its distinctive sensory and bioactive profile. Its qualitative attributes derive from a complex mixture of secondary metabolites, in particular crocins, which are highly water-soluble carotenoid glycosides that confer intense coloration, picrocrocin, a monoterpene glycoside that confers the characteristic flavor, and safranal, a monoterpene aldehyde responsible for the particular aroma [1,2,3,4,5]. In addition to their organoleptic contributions, these compounds have been extensively documented in the scientific literature for their diverse biological activities, broad potent antioxidant and radical-scavenging capabilities, anti-inflammatory effects via modulation of cytokine expression, neuroprotective actions linked to inhibition of neuronal apoptosis and oxidative stress pathways, and antiproliferative or pro-apoptotic effects against a variety of cancer cell lines [6,7,8,9]. Such multifunctional bioactivity supports the growing interest in saffron as a functional ingredient in nutraceutical, pharmaceutical, and cosmetic applications [10,11,12,13,14,15]. Despite its well-documented bioactivity, the incorporation of saffron into functional food, nutraceutical, and pharmaceutical products is substantially constrained by the pronounced chemical and physicochemical instability of its principal apocarotenoids [16,17,18,19,20] and by their scarce absorption into the circulation when orally administered [21,22]. Crocins, which are glycosylated derivatives of the carotenoid crocetin, are particularly disposed to rapid degradation when exposed to light, elevated thermal conditions, and oxidative environments; these conditions induce processes that induce cleavage of conjugated double bonds, loss of glycosidic moieties, and subsequent diminishment of biological functionality. Such instability can occur during processing, storage, and even gastrointestinal transit [23], thereby limiting the efficacy of saffron-derived formulations. In response to these challenges, a range of advanced encapsulation strategies, including nano- and micro-particulate delivery systems, have been investigated with the dual aim of physically shielding crocins from environmental stressors and enabling controlled, site-specific release of the bioactives to target tissues or absorption sites [17,24]. Liposomes, self-assembled vesicular systems consisting of one or more concentric phospholipid bilayers enclosing an aqueous core, represent a versatile and scientifically validated nanocarrier platform for the delivery of saffron-derived bioactives. Their structural architecture enables the simultaneous encapsulation of hydrophilic constituents within the aqueous interior and lipophilic molecules within the hydrophobic bilayer domains, thereby accommodating the amphipathic chemical profile of saffron’s secondary metabolites. Beyond their inherent biocompatibility and biodegradability, liposomes can enhance the physicochemical stability, protect against oxidative and photodegradation, and modulate the gastrointestinal fate of encapsulated apocarotenoids, ultimately improving their oral bioavailability and therapeutic potential [25,26,27]. Among various preparation techniques, the reverse-phase evaporation (RPE) method is particularly advantageous in hydrophilic compound encapsulation because it produces large unilamellar vesicles with high internal aqueous volume, thereby achieving superior encapsulation efficiencies for polar, crocin-rich extracts compared to many conventional methods. The main objective of this investigation was to systematically isolate and purify saffron-derived bioactive compounds through a two-step protocol consisting of an optimized hydroalcoholic extraction, followed by solid-phase extraction (SPE) for matrix cleanup and enrichment of apocarotenoid fractions. A critical aspect in SPE fractionation is the optimization of elution conditions to ensure the efficient recovery of crocins while minimizing solvent use and avoiding the unnecessary dilution of the fractions. Traditional univariate optimization is labor-intensive and may overlook factor interactions. In contrast, the use of Design of Experiments (DoE) provides a systematic and statistically robust approach to investigate the effect of multiple variables simultaneously, identifying not only the main effects but also possible curvatures or interactions [28,29]. In this study, a two-factor, three-level design was applied to optimize the elution of crocins in the second SPE fraction, focusing on methanol percentage and elution volume.

The attainment of standardized extracts can be useful in further biological investigation to clarify the role of each class of main components of saffron and the importance of synergistic effect and consequently to identify the most efficacious fraction from the therapeutic point of view. The purified extract was subsequently subjected to HPLC characterization to confirm the retention of its principal bioactive constituents, with an emphasis on crocins and picrocrocins content. The information obtained could be useful for saffron authentication and fraud detection. Then, the extract was incorporated into liposomal delivery systems prepared via RPE employing an unsaturated (UN-PC) or saturated (SAT-PC) lipid matrix extracted by soy in order to examine the influence of bilayer physicochemical properties on encapsulation performance. Quantitative assessments of encapsulation efficiency (EE) and recovery yield were performed under rigorously controlled analytical conditions, enabling comparative evaluation of formulation behavior and the elucidation of lipid-composition-dependent effects on the retention and stability of the encapsulated saffron apocarotenoids.

## 2. Results

### 2.1. Extraction and SPE Fractionation

**Figure 1:**
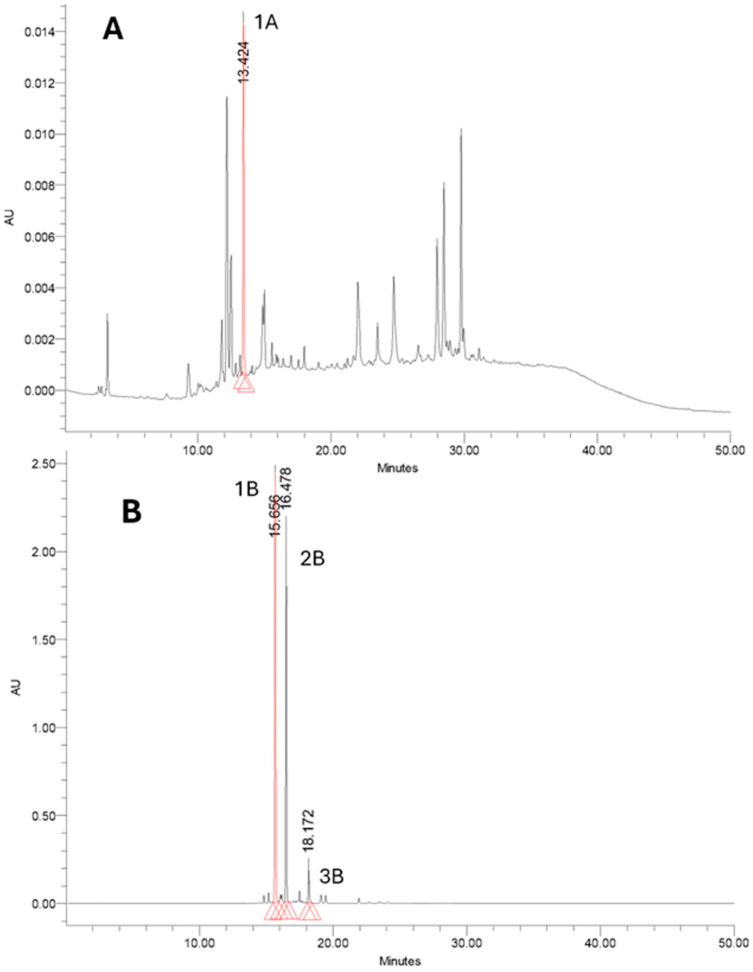
Solid-phase extraction (SPE) fractionation of saffron aqueous extract. (**A**) Chromatogram extracted at 250 nm of the first fraction (85% water, 15% methanol), enriched in picrocrocins (1A), and devoid of crocins. (**B**) Chromatogram extract at 440 nm of the second fraction (20% water, 80% methanol), enriched in crocins: 1B trans-4-GG; 2B trans-3-Gg; 3B cis-4GG, lacking picrocrocins. Retention times are shown on the chromatograms.

Hydroalcoholic extraction of finely ground saffron stigmas using an ethanol/water mixture (1:1, *v*/*v*) produced an intensely colored orange-red solution, visually indicative of a high crocin content. This extract, after clarification by low-speed centrifugation to remove particulate matter and subsequent membrane filtration, was subjected to solid-phase extraction (SPE) employing Oasis HLB cartridges as a purification step. The SPE process was conducted using a programmed sequence of methanol–water eluents of increasing organic content, which facilitated the selective removal of highly polar co-extractives such as sugars, proteins, and phenolic acids, while promoting the retention and recovery of crocin-rich fractions. Each elution step was performed with a volume of 4 mL solvent. Quantitative analysis of the recovered eluates indicated that the SPE protocol achieved an average crocin recovery of 85.4 ± 1.8% (n = 3), underscoring both the reproducibility and the efficiency of the method in isolating and preserving target apocarotenoids from saffron without significant degradation or loss. Importantly, the first elution fraction, obtained with 85% water and 15% methanol, was enriched in picrocrocins and contained no detectable crocins (Figure 1A), while the second fraction, recovered with 20% water and 80% methanol, was selectively enriched in crocins, confirming the high selectivity of the SPE procedure (Figure 1B).

To further optimize the recovery of crocins in the second elution, a two-factor, three-level factorial design (DoE) was applied; the results are summarized in Table 1.

**Figure 2:**
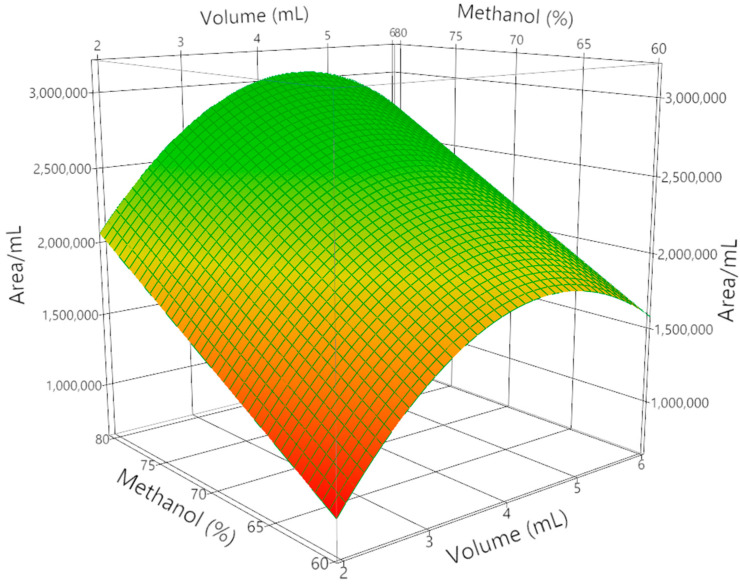
Response surface plot showing the effect of elution volume (mL) and methanol percentage (%) on crocin recovery, expressed as the chromatographic area of *trans*-4GG normalized per mL of solvent.

The effects of elution volume (2, 4, and 6 mL) and methanol content in water (60, 70, and 80%) were investigated, using the chromatographic area of a representative crocin (*trans*-4GG) normalized per mL of solvent as the response variable. Analysis of variance (ANOVA) demonstrated that the model was highly significant (F = 17.74, *p* = 0.0012), with a determination coefficient R^2^ of 0.88 and adjusted R^2^ of 0.83, confirming the reliability of the regression. Lack-of-fit was not significant (*p* = 0.1636), supporting the model validity. Both methanol percentage and elution volume exerted significant linear effects on crocin recovery (*p* < 0.05), with methanol percentage being the dominant factor (t = 5.46, *p* = 0.0009). Moreover, the quadratic term for elution volume was significant (t = –3.68, *p* = 0.0078), revealing a curvature in the response: at low volumes (2 mL), crocins were only partially desorbed from the SPE material; at intermediate volumes (around 4 mL), recovery was maximized due to efficient desorption without excessive dilution; meanwhile, at higher volumes (6 mL), the normalized response decreased because of dilution effects as shown in Figure 2.

Overall, the DoE confirmed that crocin recovery was maximized under intermediate volumes and higher methanol percentages, identifying 70–80% methanol and approximately 4 mL of eluent as optimal conditions. These findings demonstrate that the combination of SPE fractionation with statistical optimization provides a robust and reproducible approach to selectively isolate crocins and picrocrocins from saffron extracts while minimizing solvent consumption.

### 2.2. Characterization of Saffron Extract

The purified saffron extract was characterized by high-performance liquid chromatography (HPLC) equipped with a diode array detector (DAD), ensuring both qualitative and quantitative assessment of the bioactive profile. Chromatographic separation confirmed that crocins were the predominant constituents, displaying an intense absorption maximum at 441 nm. Distinct peaks at 250 nm were assigned to the bitter monoterpene glycoside picrocrocin, while additional signals attributable to the volatile aldehyde safranal were also observed. Critically, the two-step SPE fractionation protocol provided a clear partitioning of the aqueous extract: the first fraction, eluted with 85% water and 15% methanol, was enriched in highly polar metabolites such as picrocrocin, and its chromatogram showed no detectable crocin signals; conversely, the second fraction, obtained with 20% water and 80% methanol, concentrated less polar apocarotenoids such as crocins as confirmed by chromatographic analysis. The absence of crocin peaks in the first fraction and the absence of picrocrocin peaks in the second confirmed the high selectivity of the SPE protocol. Furthermore, the SPE step reduced the levels of interfering matrix constituents such as polysaccharides, proteins, and phenolics. This purification improved the reliability of quantitative HPLC characterization, ensuring that the recovered fractions were analytically reproducible. Consequently, the subsequent liposomal encapsulation experiments were carried out using extracts of sufficient purity, ensuring that the bioactives incorporated into liposomes were chemically well-defined and free from interfering impurities. Working with such extracts is essential for accurately assessing the effects of encapsulation and their biological activity in further experiments, thereby improving the interpretability of bioavailability, efficacy and delivery studies. In fact, it is well known from the literature that the chemical composition of saffron and its therapeutic properties are strictly related [30,31].

### 2.3. Liposomes Characterization and Entrapment Efficiency

**Figure 3:**
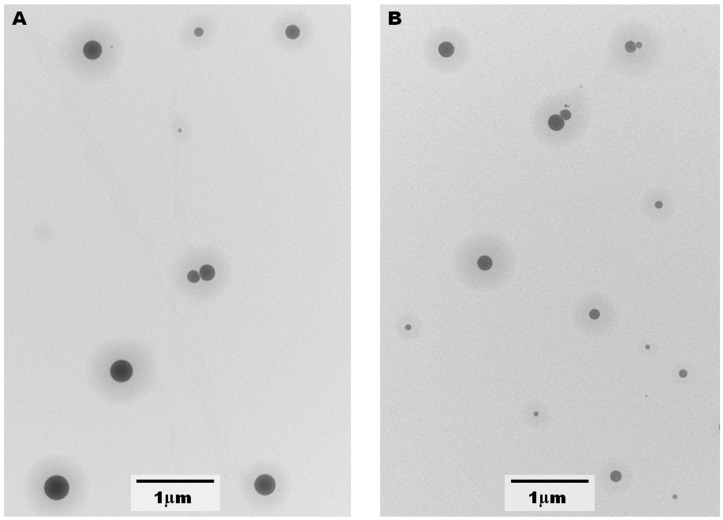
TEM images of 10 mM (**A**) UN-PC liposomes and (**B**) SAT-PC liposomes soon after their preparation.

The saffron extract was entrapped in liposomes to increase its stability and to enhance its uptake at intestinal level. In this preliminary investigation, we used soy-derived lipid-saturated or -unsaturated mixtures to evaluate the role of lipid packing and fluidity on the ability of the aggregates to entrap and retain the extract. RPE was chosen in this investigation because it is well known that this technique makes it possible to obtain stable aggregates and high EE in cases of water-soluble compounds. Nanoparticle Tracking Analysis (NTA) analysis showed that liposomes featured a monodisperse population centered about 200 nm and stable for at least 3 months. These results are in agreement with what can be observed in the Transmission Electron Microscopy (TEM) images, as reported in Figure 3 for the liposomes analyzed soon after their preparation. TEM measurements repeated after 3 months gave similar results.

The three most abundant components of saffron extract were entrapped with different efficiency in liposomes: the inclusion of *cis*-4GG was almost quantitative (EE > 90%), while the EE for *trans*-4GG was slightly higher than 50%, independently from the lipid used for their preparation. The lowest EE and dependence from the lipid used for liposomes preparation was observed for picrocrocins: 20% for SAT-PC and about 5% for UN-PC. Overall, these results suggest that EE and polarity of the loaded molecules are inversely proportional. It is reasonable to hypothesize that the polarity of the entrapped molecules plays a key role during liposomes preparation applying reverse-phase evaporation. In fact, it is well known that, according to this procedure, before liposome formation, inverse micelles are present in the water-in-oil emulsion. According to the literature, the polarity of the organic solvent used affects the size of the inverse micelles and the intermediate aggregates that form during liposomes preparation because of the interaction between the lipid and the medium (organic solvent and water): the different polarities affect the orientation and organization of the lipids in inverse micelles. Obviously, also the polarity of lipids and of the molecules to be included in liposomes affects physicochemical interactions between the solvents and liposomes components (either lipids and liposomes cargo) [32]. It is thus evident that the polarity of the molecules present in saffron extract can affect liposomes entrapment efficiency. In particular, cis-4GG molecules, being less polar, will partition to a higher extent in the organic phase of the emulsion formed during liposomes preparation; meanwhile, picrocrocins, the most polar, will concentrate in the aqueous phase of inverse micelles. It is possible that, upon bilayer formation, part of the molecules present in the organic phase will be embedded in the liposome bilayer, thus increasing their overall entrapment efficiency. The fact that doubling the concentration of the extract hampers the formation of liposomes supports this hypothesis. It cannot be excluded that the size also affects the ability of the formulation to entrap the molecules contained in the extract: picrocrocins are smaller molecules with respect to crocins; so, despite their higher polarity, they could escape the bilayer, especially in the case of UN-PC liposomes (as suggested by EE values) characterized by a high fluidity of the bilayer. On the other hand, crocins, besides being more bulky (especially *cis* one) than picrocrocins, have two disaccharide units that cannot freely cross the bilayer. In particular, it is known from the literature that disaccharides strongly interact with the phospholipid bilayer through multiple hydrogen bonds arriving, at a high concentration, to replace hydration shell water molecules, leaving the overall structure of the bilayer undisturbed [33,34]; meanwhile, only a few monosaccharide molecules can interact with the bilayer membrane [35]. It reasonable to hypothesize that the different ability to interact with polar lipid headgroups plays a crucial role in the observed EE: according to the RPE technique, prior to liposome formation, the system passes through an emulsion phase and then through a gel phase, starting from inverse micelles. At a specific concentration during slow organic solvent removal, the gel collapses and the lipids left in the solution form a bilayer, arranging themselves around the inverted micelles that include the liposomes’ aqueous pool [36]. It is thus evident that the effect and the extent of the specific interaction between lipids and the different molecules included in the aggregates can vary significantly in the different phases of this multistep procedure. During liposomes preparation, we noticed that the emulsion obtained after bath sonication was particularly unstable for both formulations tested, so we decided to evaporate the organic solvent using the rotary evaporation as described in Section 3.4; however, we immersed the round-bottom flask in the bath sonicator at 30 °C. We did not observe differences in liposomes properties in terms of size, stability, or EE. This evidence suggests that the slowness with which the organic solvent is removed is more important than the stability of the emulsion obtained after bath sonication.

## 3. Materials and Methods

### 3.1. Materials

Dried saffron (*Crocus sativus* L.) stigmas of certified geographical origin and premium-grade quality were sourced from a local producer under controlled storage conditions to minimize exposure to light, heat, and humidity prior to analysis. Analytical-grade solvents—including ethanol, methanol, acetonitrile, methyl t-butyl ether, chloroform and ethyl acetate—of HPLC purity were procured from Sigma-Aldrich (St. Louis, MO, USA) and Milli-Q ultrapure water produced with a Millipore purification system (resistivity 18.0 MΩ·cm, Darmstadt, Germania). Solid-phase extraction was performed using Oasis HLB cartridges (3 cc; Waters Corporation, Milford, MA, USA) packed with a hydrophilic–lipophilic balanced copolymer sorbent, suitable for retaining polar apocarotenoids. Two phospholipid types were selected for liposome formulation: L-α-phosphatidylcholine from soy lecithin with ≥95% purity (UN-PC) and fully hydrogenated soybean phosphatidylcholine enriched in C18-saturated fatty acyl chains (SAT-PC), both obtained from Avanti Polar Lipids (Alabaster, AL, USA) and stored under an inert atmosphere at −20 °C until use to preserve lipid integrity.

### 3.2. Extraction and SPE Purification

Saffron stigmas (50 mg) were finely ground using a porcelain mortar and pestle to ensure a uniform particle size, thereby maximizing solvent–solid contact during extraction. The powdered stigmas were quantitatively transferred to a glass vessel and extracted with 10 mL of a hydroalcoholic mixture of ethanol and deionized water (1:1, *v*/*v*) under continuous magnetic stirring for 60 min at ambient temperature (22 ± 1 °C), with the vessel wrapped in aluminum foil to protect light-sensitive apocarotenoids. Following extraction, the suspension was centrifuged at 1000 rpm for 5 min to sediment-insoluble plant material, and the supernatant was carefully decanted and passed through a 0.45 μm nylon membrane filter to remove residual particulates. Solid-phase extraction (SPE) was performed to fractionate picrocrocins and crocins. For this purpose, 5 mL of saffron extract was diluted with 35 mL of water and loaded onto Oasis HLB cartridges (3 cc), previously conditioned with 6 mL of methanol and 3 mL of water. This dilution step reduced both viscosity and residual organic strength of the extract, ensuring complete retention of crocins on the HLB phase. In preliminary tests, undiluted loading led to partial crocin breakthrough in the first fraction; dilution restored a fully aqueous environment compatible with selective SPE trapping before elution. Preliminary trials with different methanol/water ratios were conducted to identify suitable elution conditions. The optimized protocol consisted of two sequential elutions: the first with 85% water and 15% methanol, yielding the picrocrocin-rich fraction, and the second with 20% water and 80% methanol, yielding the crocin-rich fraction. Between the two steps, cartridges were washed with 1 mL of water to remove residual impurities. In order to optimize the recovery of crocins in the second elution, a two-factor, three-level factorial design (DoE) was applied, considering elution volume X_1_ (2, 4, and 6 mL) and methanol content in water X_2_ (60, 70, and 80%) as independent variables, as reported in Table 2.

The response variable was defined as the chromatographic area of a representative crocin (*trans*-4GG), normalized per mL of eluent. Eleven experimental runs were performed according to the factorial design, and the resulting eluates were analyzed by HPLC-DAD, as described in Section 3.3.

### 3.3. HPLC Analysis

For the analysis of the aqueous saffron extract, a Waters 600 HPLC system (Milliford, MA, USA) equipped with an Agilent Technologies 1220 degasser (Waldbronn, Germany), an autosampler Waters 717 plus, and a Security Guard Ultra UHPLC C18 (4.6 mm i.d., Phenomenex, Torrance, CA, USA) pre-column coupled to a Kinetex C18 analytical column (250 mm × 4.6 mm, 5 μm particle size, Phenomenex) was employed. Chromatographic separation was carried out using a gradient elution program with water (A) and acetonitrile (B) as the mobile phases. The mobile phase was delivered at a flow rate of 1.0 mL·min^−1^. Initial conditions were set at 5% B and 95% A, reaching 95% B within 30 min, and returning to initial conditions within 20 min for a total run time of 50 min. Detection was performed using a PDA 996 photodiode array detector (Waters), and data acquisition and processing were managed with Empower software (Waters). Identification of crocins and picrocrocin was achieved by comparison of their retention behavior and UV–Vis spectral profiles with those reported in the literature for saffron apocarotenoids [37,38,39]. The diagnostic absorption maxima at 441 nm for crocins and at 250 nm for picrocrocin, combined with the agreement of chromatographic and spectral features with published datasets, allowed an unambiguous assignment in the absence of suitable pure commercial standards.

### 3.4. Liposome Preparation

A proper amount of lipid (23.6 mg of UN-PC or 24.2 mg of SAT-PC) was solubilized in CHCl_3_ in a round-bottom flask; then, the solvent was removed using a rotary evaporator. The lipid film was left under reduced pressure for at least 2 h; then, organic solvent (2 mL methyl t-butyl ether and 1 mL chloroform for SAT-PC and 3 mL ethyl acetate for UN-PC) and 1 mL of ultrapure water (in the case of void liposomes) were added to the round-bottom flask. To prepare loaded liposomes, the extract, prepared as described in Section 3.2, was diluted 4 times after nylon membrane filtration and was added to the lipid film together with the organic solvent instead of ultrapure water. The obtained two-phase solution was emulsified using a bath sonicator for 5 min at room temperature and then the organic solvent was slowly removed using a rotary evaporator: the temperature was set to 30 °C and the pressure was reduced to 50 mbar every 15 min, starting from 350 mbar until 150 mbar; this was then kept for 5 min at this reduced pressure. At this point, 2 mL of ultrapure water was added, the dispersion was vortex-mixed, and the mixture was kept in a rotary evaporator at 130 mbar for 10 min to obtain a homogeneous suspension. The final lipid concentration was 10 mM.

### 3.5. Liposome Dimensions, Stability, and Morphology

Particle size and particle size distribution were assessed by NTA using Nanosight NS300 (Malvern Panalytical Ltd, Malvern, UK) equipped with a blue laser (λ = 488 nm). Liposomes were diluted with Milli-Q ultrapure water up to 4 × 10^−6^ M total lipid concentration and were introduced into the instrument cell utilizing a syringe pump (flow rate of 50 μL/s). Five acquisitions that captured 25 frames/s (60 s in total) were performed. The same measurements were carried out every 2 weeks for 3 months to evaluate liposome stability. The morphology of the aggregates was investigated by Transmission Electron Microscopy (TEM) using a Philips CM100 TEM microscope (Philips/FEI Company, Eindhoven, Paesi Bassi): 10 μL of 10 mM liposome suspension was air-dried onto a copper grid and analyzed.

### 3.6. Encapsulation Efficiency (EE)

After liposomes preparation, unentrapped extract was removed through centrifugation (36,000× *g*, 4 °C, 1 h). The use of low temperature during centrifugation was essential to minimize thermally induced destabilization, aggregation, or leakage of encapsulated bioactive compounds. Liposomes suspension before centrifugation and the supernatant obtained by centrifugation (containing the unentrapped extract) were analyzed by HPLC analysis, as described in Section 3.3. The percentage ratio between the intensity of the signal observed for picrocrocin or crocins (taking into account the dilution) and unfiltered liposomes suspension at the proper wavelength allowed us to obtain the EE, calculated according to Equation (1):

EE% = [(Total analyte − Free analyte)/Total analyte] × 100,(1)

where analyte concentration was determined for each target compound based on the integrated HPLC peak areas.

## 4. Conclusions

This study demonstrates that saffron metabolites can be efficiently extracted, purified, and fractionated in order to separate the main different classes of compounds present in the extract. This result will enable us to test the fractions on cell cultures with the aim of identifying the most active classes of compounds, deepening our knowledge on the synergic effect in saffron extract. The encapsulation of the extract into liposomes evidenced that, when using the RPE method, the polarity of the loaded molecules plays a key role in the extent of their encapsulation inside liposomes. The use of SoyPC95 lipids resulted in higher encapsulation efficiency compared with hydrogenated C18 phosphatidylcholine, underlining the role of bilayer fluidity and composition on encapsulation performance. Beyond improving physicochemical stability, liposomal encapsulation is also expected to protect apocarotenoids from oxidative, gastrointestinal, and thermal degradation, extending their shelf-life, and, according to the literature, enhancing intestinal absorption [25,26,27]. Beyond protecting apocarotenoids from oxidative and gastrointestinal degradation and extending their shelf-life, liposomal encapsulation is also expected to enhance intestinal absorption, supporting their effective delivery to target tissues. These benefits are highly relevant not only for nutraceutical and functional food applications, but also for advanced pharmaceutical formulations, where liposomes are widely employed as vehicles for controlled drug delivery and targeted release. These highly relevant benefits could be extended to other nutraceutical and/or functional food applications: the extract of saffron crocus by-products such as tepals (an abundant low-cost material known for their interesting biological activity [20,40] could be included in liposomes to assess and eventually exploit their therapeutic potential while valorizing an agri-food waste product. Future investigations should therefore expand toward mixed liposomes to evaluate the effect of lipid components on the physicochemical properties and biological behaviors of the aggregates; it is possible to use in vitro digestion and bioaccessibility assays and ultimately in vivo evaluations to confirm the therapeutic potential of saffron-loaded liposomes in biomedical contexts.

## Figures and Tables

**Table 1:** Regression coefficients and ANOVA summary for the factorial design applied to optimize crocin recovery in the SPE elution. Reported values correspond to regression coefficients (estimate ± standard deviation), model fit indices (R^2^ and adjusted R^2^), and analysis of variance (ANOVA) with significance levels for each source of variation. The response variable was defined as the chromatographic area of *trans*-4GG crocin normalized per mL of eluent. Terms marked with an asterisk (*) are statistically significant (*p* < 0.05).

Parameters	Value ± SD		R^2^	Adj-R^2^	
intercept	2.41 × 10^6^ ± 0.11 × 10^6^				
* X_1_	3.2 × 10^5^ ± 1.0 × 10^5^				
* X_2_	5.6 × 10^5^ ± 1.0 × 10^5^		0.883	0.834	
* X_1_^2^	-5.4 × 10^5^ ± 1.5 × 10^5^				
variation source	sum of squares	degrees of freedom	mean square	F-value	*p*-value
lack of fit	4.1 × 10^11^	5	8.3 × 10^10^	5.4001	0.1636
pure error	3.1 × 10^10^	2	1.5 × 10^10^		
model	3.4 × 10^12^	3	1.1 × 10^12^	17.7387	0.0012
residual	4.5 × 10^11^	7	6.4 × 10^10^		

**Table 2:** Experimental matrix of the two-factor, three-level factorial design (DoE) applied to the optimization of crocin recovery in the second SPE elution. The response variable was the chromatographic area of a representative crocin (*trans*-4GG) normalized per mL of solvent. Runs marked with an asterisk (*) are replicates.

Run	Volume [mL]	CH_3_OH [%]	X_1_	X_2_	Area Crocin/Volume [AU/mL] × 10^3^
1	2	60	−1	−1	1453
2	2	70	−1	0	1950
3	2	80	−1	1	2250
4	4	60	0	−1	2250
5	4	70	0	0	3091
6	4	80	0	1	3284
7	6	60	1	−1	1750
8	6	70	1	0	1928
9	6	80	1	1	2226
10 *	4	70	0	0	3091
11 *	4	70	0	0	3064

## Data Availability

Data is available within the article.

## Abbreviations

The following abbreviations are used in this manuscript:

RPE	reverse-phase evaporation
SPE	solid-phase extraction
DoE	Design of Experiments
UN-PC	unsaturated lipid matrix extracted by soy
SAT-PC	saturated lipid matrix extracted by soy
EE	encapsulation efficiency
ANOVA	analysis of variance
HPLC	high-performance liquid chromatography
DAD	diode array detector
NTA	Nanoparticle Tracking Analysis
TEM	Transmission Electron Microscopy
